# Swedish snuff (snus) dipping, cigarette smoking, and risk of peripheral artery disease: a prospective cohort study

**DOI:** 10.1038/s41598-022-16467-x

**Published:** 2022-07-15

**Authors:** Shuai Yuan, Olga E. Titova, Scott M. Damrauer, Agneta Åkesson, Susanna C. Larsson

**Affiliations:** 1 Unit of Cardiovascular and Nutritional Epidemiology, Institute of Environmental Medicine, Karolinska Institutet, Nobelsväg 13, 17177 Stockholm, Sweden; 2 Unit of Medical Epidemiology, Department of Surgical Sciences, Uppsala University, Uppsala, Sweden; 3 Corporal Michael J. Crescenz VA Medical Center, Philadelphia, PA USA; 4 Department of Surgery, University of Pennsylvania Perelman School of Medicine, Philadelphia, PA USA

**Keywords:** Cardiology, Risk factors

## Abstract

Tobacco smoking is an important risk factor for peripheral artery disease (PAD), but it remains unknown whether smokeless tobacco, such as Swedish snuff (snus), is also associated with this disease. We used data from the Cohort of Swedish Men including 24,085 men. Individuals were grouped into never, past, and current snus dippers as well as never, past quitting ≥ 10 years, past, quitting < 10 years, and current smokers. Incident PAD cases were defined by linkage of the cohort with the Swedish National Patient Register. Cox proportional hazards regression was used to analyze the data. Over a mean follow-up period of 9.1 years (from July 1, 2009 to December 31, 2019), 655 incident PAD cases were ascertained. Cigarette smoking but not Swedish snus dipping was associated with an increased risk of PAD. Compared with never snus dippers, the hazard ratio of PAD was 0.95 (95% confidence interval [CI] 0.73–1.24) for past snus dippers and 0.88 (95% CI 0.66–1.17) for current snus dippers. Compared to never smokers, the hazard ratio of PAD was 1.38 (95% CI 1.14–1.68) for past smoker who stopped smoking for ≥ 10 years, 2.61 (95% CI 1.89–3.61) for past smoker who stopped smoking for < 10 years, and 4.01 (95% CI 3.17, 5.08) for current smoker. In conclusion, cigarette smoking but not Swedish snus dipping increases the risk of PAD.

## Introduction

Peripheral artery disease (PAD) is a common atherosclerotic cardiovascular disease with a global prevalence of 5.6%^1^. It was estimated that PAD affected around 237 million individuals aged above 25 years worldwide in 2015^1^. Traditional cardiovascular risk factors, like smoking, diabetes, dyslipidemia, and hypertension, and several metabolic and inflammatory factors have been associated with PAD risk in populations in Western countries^2^. Among these factors, tobacco smoking is an important modifiable risk factor for PAD^3^, and the causality of the association has been strengthened in recent Mendelian randomization studies^4,5^. Nicotine is one of the detrimental chemicals in tobacco and has the potential of impairing cardiovascular system via unfavorable impacts on heart rate, blood lipids, blood pressure, blood viscosity, and vasoconstriction as well as on endothelial function^6–8^.

Oral snuff is a smokeless tobacco product containing nicotine but limited other detrimental components from cigarette smoking. Studies have suggested that snuff dippers are exposed to similar or higher doses of nicotine than smokers^9^. Snuff in different countries and areas may be largely varying in addiction potential and patterns of usage^10^ and have different impacts on cardiovascular disease^11^. In Sweden, approximately 11–13% of the population used Swedish snuff (snus) and 7–12% of the population smoked daily in a recent decade^12^. Previous studies on snus revealed that snus dipping was not associated with risk of overall heart disease^13^, stroke^14^, acute myocardial infarction^15^ or ischemic heart disease^16^ but associated with an increased risk of cardiovascular mortality^17^. However, limited data are available on the effect of Swedish snus dipping on PAD risk. Here, we aimed to explore the association between snus dipping and incident PAD using data from a cohort of middle-aged and older men. We also examined the association for tobacco smoking as a positive control.

## Materials and methods

### Study population

**Figure 1:**
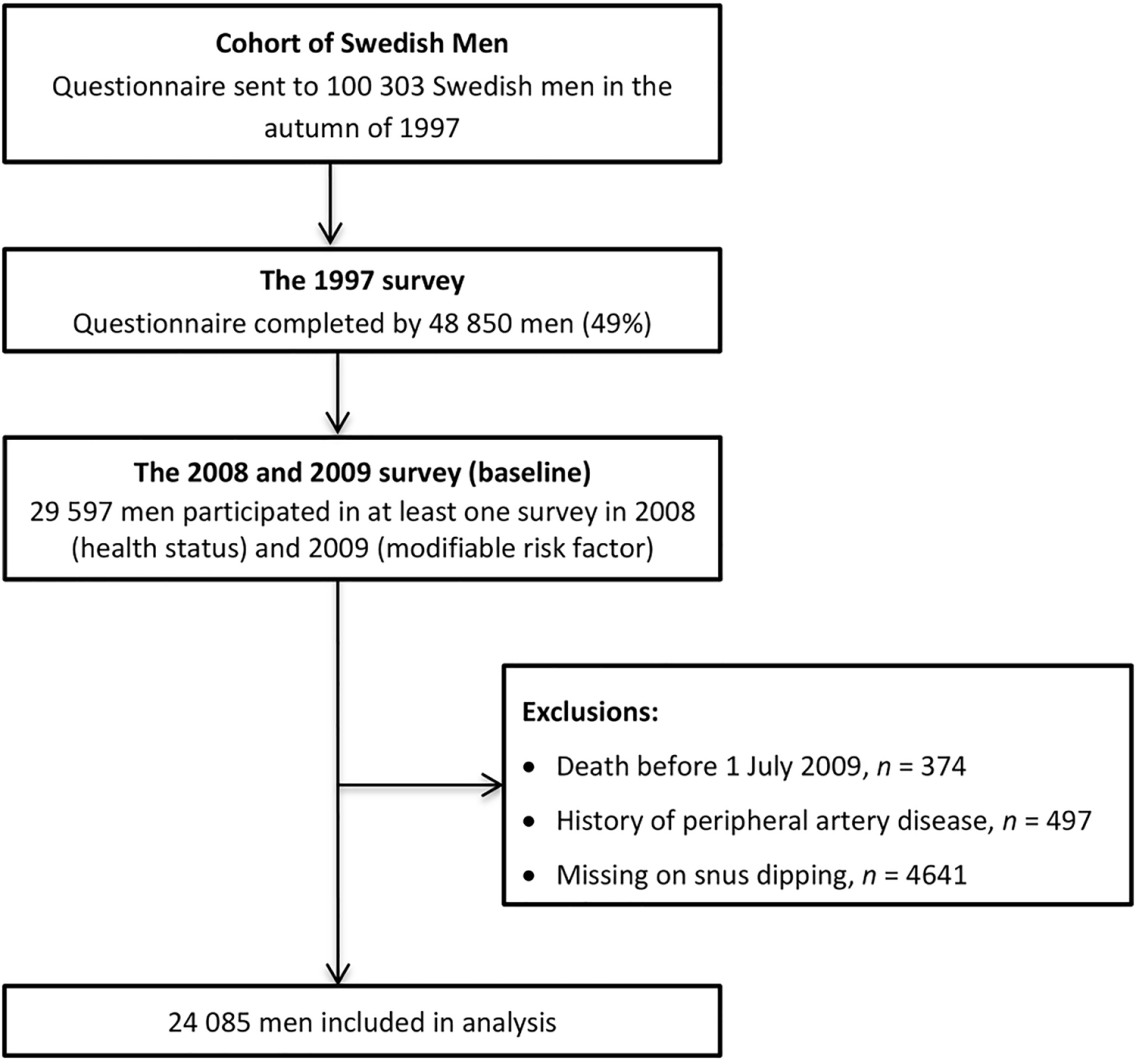
Flow chart of the final study population.

We included individuals from the Cohort of Swedish Men (COSM) that was initiated in the late autumn of 1997 by inviting 100,303 Swedish men aged 45 to 79 living in Västmanland or Örebro county^18^. Around 49% of invited individuals (48,850) responded the questionnaire in 1997. The second surveys were conducted in 2008 and 2009 when 37,861 and 29,068 COSM members who were still alive and residing in the study areas received questionnaires about health status (2008) and modifiable risk factors (2009), respectively. Around 78% (29,531) and 90% (26,161) of men completed the 2008 and 2009 questionnaires, respectively. A total of 29,597 men participated in at least one survey in 2008 and 2009. After removal of individuals who died before July 1, 2009, those with a prior diagnosis of PAD, and those with missing information on snus dipping (in the analysis of snus dipping), 24,085 men were included in the analysis. The flow chart of the final study population is presented in Fig. 1. Data used in this study were permitted under Project simpl2020002. The study has been approved by the Swedish Ethical Review Authority (Dnr: 2019-03986). All participants have provided informed consent. The research was performed in accordance with the Declaration of Helsinki.

### Assessment of snus dipping and tobacco smoking

Snus dipping and tobacco smoking were measured by a self-administrated questionnaire in 2009. Participants were asked to answer the question “mark if you have used snus regularly (more than 5 servings per week)” by choosing one of three predefined categories, including “No, I have never used snus regularly”, “Yes, I use snus” and “Yes, but I stopped using snus”^19^. Three statuses (never, past, and current snus dippers) were defined based on this question. Likewise, tobacco smoking was defined using corresponding information on “mark if you have smoked cigarettes regularly (more than 5 cigarettes per week)”. Four categories (never, past with quitting time ≥ 10 years, past with quitting time < 10 years and current smoker) were defined when taking time of stopping smoking into consideration. Individuals without smoking information (n = 1172) were grouped into one category named “missing” in the analysis. Never snus dipper and never smoker were defined as the reference groups in the analysis of snus dipping and tobacco smoking, respectively, in relation to PAD risk.

### Ascertainment of cases and follow-up

Incident PAD cases were ascertained by the clinical diagnosis based on codes from the 9th (440.0, 440.2, 440.3, 440.4, 440.9, 443.9) and 10th (I70.0, I70.2, I70.3, I70.4, I70.5, I70.6, I70.7, I70.9, I703.9) International Classification of Disease revision with information identified by linkage of the cohorts to the Swedish Patient Register. The register has an nearly complete coverage of hospital-based inpatient and outpatient care^20^. Individuals were followed up from July 1, 2009 until the date of diagnosis of PAD, date of death, or end of follow-up (i.e., 31 December 2019), whichever came first. Death information was obtained from the Swedish Death Registry.

### Assessment of covariables

Information on age, body mass index, highest education attainment, history of hypertension, hypercholesterolemia, and diabetes mellitus, physical activity (walking/cycling and exercise combined), diet quality (measured by modified Dietary Approaches to Stop Hypertension) and coffee consumption was reported in the 2008 and 2009 questionnaires. The modified Dietary Approaches to Stop Hypertension diet included fruits, vegetables, nuts and legumes, whole grains, and low‐fat dairy products as healthy components and red and processed meat and sweetened beverages as unhealthy components^21^. Individuals were assigned a score from 1 to 5 according to the quintiles of consumption of each food and the scores were summed to create a diet score (7–35). A high score indicates a high adherence to the modified Dietary Approaches to Stop Hypertension diet pattern.

### Statistical analysis

The Kaplan–Meier method and log-rank test were used to compare the probability of being free of PAD diagnosis across snus dippers and cigarette smokers. Cox proportional hazard regression was used to estimate the associations of snus dipping and tobacco smoking with risk of incident PAD with age as the underlying time scale. The assumption of proportionality was verified using Schoenfeld residuals. We obtained estimates from an age-adjusted model and a multivariable-adjusted model with adjustment for age, body mass index (underweight, normal, overweight and obesity), education levels (≤ 9, 10–12, > 12 years), history of hypertension, hypercholesterolemia, and diabetes mellitus (yes or no), tobacco smoking in the analysis of snus dipping, snus dipping in the analysis of tobacco smoking, physical activity (0–10, 11–30 and 31–60 and > 60 min per day), and diet score (continuous). The proportion of missing data was 3.32% for body mass index, 0.24% for education level, 16.9% for smoking status, and 11.9% for physical activity. A separate group was created for each variable with missing values. All statistical tests were two-sided, and the analyses were performed in Stata/SE (version 15.0; StataCorp, Texas, USA). An association with a *p* value below 0.05 was deemed as statistically significant.

## Results

The mean age of the population at baseline was 70.2 ± 8.3 years. A total of 655 incident PAD cases were ascertained during a mean follow-up period of 9.1 years and 220,127 person-years (from July 1, 2009 to December 31, 2019). The age of PAD onset was 78.2 ± 8.0 years, and the incidence rate was 3.0 per 1000 person-years. The age-standardized baseline characteristics by snus dipping are displayed in Table 1. Snus dippers were more likely to be younger men with low education level, low physical activity, and with a history of hypertension, hypercholesterolemia, diabetes, and past or current cigarette smokers.

**Table 1:** Age-standardized baseline characteristics of 24,085 Swedish men by snus dipping status.

Characteristics	Snus dipping			Total
	Never	Past	Current	
Individuals	18,789	2946	2350	24,085
Age, mean ± SD, years	71.2 ± 8.4	66.8 ± 7.0	67.0 ± 7.2	70.2 ± 8.3
Body mass index^a^, mean ± SD, kg/m^2^	25.6 ± 3.1	26.1 ± 3.1	26.0 ± 3.3	25.7 ± 3.2
Post-secondary education^a^, %	21.8	15.0	13.5	20.1
Hypertension, %	39.4	44.3	40.4	40.1
Hypercholesterolemia, %	25.3	30.8	25.1	25.8
Diabetes, %	9.6	11.2	10.5	9.8
**Smoking status**^**a**^**, %**				
Never smoker	52.0	15	16.7	44.3
Past smoker	34.8	75.2	60.3	42.3
Current smoker	8.9	5.3	11.0	8.5
**Physical activity**^**a**^**, %**				
< 10 min/day	5.7	5.2	7.1	5.8
10–30 min/day	15.4	15.1	19.6	15.7
31–60 min/day	50	48.9	48.2	49.6
> 60 min/day	27.6	29.8	23.5	27.7
DASH score, mean ± SD	18.1 ± 3.2	18.1 ± 3.1	17.3 ± 3.1	18.0 ± 3.2
Coffee consumption, servings, mean ± SD	2.8 ± 1.8	3.1 ± 2.0	3.1 ± 2.0	2.8 ± 1.9

*SD* standard deviation.

^a^Proportion of missing was 3.32% for body mass index, 0.24% for education levels, 4.89% for smoking status, and 11.9% for physical activity.

**Figure 2:**
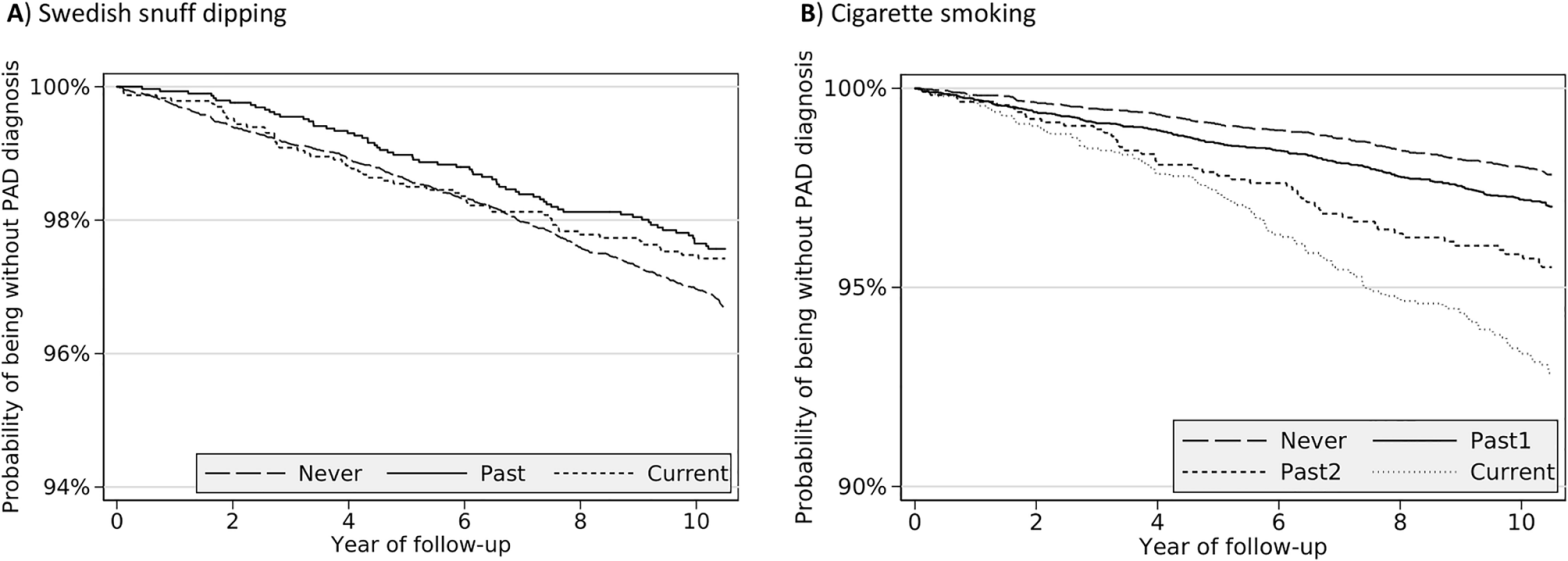
Kaplan–Meier survival curve of peripheral artery disease risk across snus dippers and smokers. Past1 indicates past smoker who stopped smoking for ≥ 10 years and Past2 indicates past smoker who stopped smoking for < 10 years.

The Kaplan–Meier survival curve showed that past snus dippers had higher probability of being free of PAD diagnosis compared to never snus dippers (Fig. 2, *p* for log-rank test = 0.02). However, snus dipping was not associated with the risk of PAD in either the age-adjusted or the multivariable-adjusted model (Table 2). Compared with never snus dippers, the multivariable-adjusted hazard ratio of PAD was 0.95 (95% confidence interval (CI) 0.73, 1.24) for past snus dippers and 0.88 ((95% CI 0.66, 1.17) for current snus dippers.

**Table 2:** Associations of tobacco smoking and snus dipping with risk of peripheral artery disease.

Group	Cases	Person-years	HR (95% CI)^a^	HR (95% CI)^b^
**Snus dipping**				
Never	533	169,425	Ref	Ref
Past	66	28,445	1.03 (0.79, 1.33)	0.95 (0.73, 1.24)
Current	56	22,256	1.10 (0.83, 1.45)	0.88 (0.66, 1.17)
**Tobacco smoking**				
Never	203	98,924	Ref	Ref
Past, quitting ≥ 10 years	236	82,688	1.48 (1.23, 1.79)	1.38 (1.14, 1.68)
Past, quitting < 10 years	48	10,977	2.90 (2.11, 3.98)	2.61 (1.89, 3.61)
Current	124	18,044	4.51 (3.60, 5.65)	4.01 (3.17, 5.08)

^a^Hazard ratios (HRs) were obtained from the age-adjusted model.

^b^HRs were obtained from the model adjusted for age, body mass index (underweight, normal, overweight and obesity), education levels (≤ 9, 10–12, > 12 years), history of hypertension, hypercholesterolemia, and diabetes mellitus (yes or no), tobacco smoking in the analysis of snus dipping, snus dipping in the analysis of tobacco smoking, physical activity (0–10, 11–30 and 31–60 and > 60 min per day), and diet score (continuous).

In Kaplan–Meier analysis, never smokers had higher probability of being free of PAD diagnosis compared to past or current smokers (Fig. 2, *p* for log-rank test < 0.001). Tobacco smoking also showed a robust and strong association with incident PAD in Cox regression (Table 2). Hazard ratios of PAD were 1.38 (95% CI 1.14, 1.68), 2.61 (95% CI 1.89, 3.61) and 4.01 (95% CI 3.17, 5.08) for past smoker who stopped smoking for ≥ 10 years, past smoker who stopped smoking for < 10 years, and current smoker, compared to never smoker. The direction and magnitude of associations remained stable in the analysis excluding individuals without smoking information (data not shown).

## Discussion

The present study found that cigarette smoking, but not Swedish snus dipping was associated with an increased risk of PAD in a cohort of middle-aged and older Swedish men. This is the first study examining the association between snus dipping and incident PAD.

Previous studies on snuff dipping in relation to other cardiovascular diseases showed no association with risk of atrial fibrillation or heart failure^22,23^, but reported conflicting results for myocardial infarction and stroke^11,13–16,24,25^. A recent cohort study found that Swedish snus dipping was not associated with risk of major heart and valvular diseases, abdominal aortic aneurysm, or cardiovascular mortality, but possibly with an increased risk of stroke^19^. The discrepancy in these studies may be caused by residual confounding (e.g., weight gain after starting dipping snus since snus is often used to quit smoking), varying ways of treating smoking status (adjusting for it as confounders or restricting analysis to never smokers), different baseline age, inadequate statistical power, and diverse effects of snus (nicotine) on different cardiovascular diseases. Given that obesity is not an important risk factor for PAD^2^ and that many important cofounders were adjusted for in our analyses, our findings is less likely to be biased by confounding from weight change and other lifestyle factors. Nevertheless, the association may be challenged by inadequate power caused by a small number of cases despite of a long follow-up of this study. However, we observed that the hazard ratios of PAD for past and current snus dippers were below one.

Tobacco smoking has been identified as an important modifiable risk factor for PAD and the magnitude of the association was larger than that for coronary artery disease^2–5,26^. Even though nicotine has been proposed to affect the cardiovascular systems in an unfavorable manner^6–8^, our study did not support a detrimental role of snus (rich in nicotine) dipping in PAD. Considering a comparably reduced risk of PAD in past smoker versus current smoker, the nicotine replacement therapy to help cessation appears to be a practical strategy for PAD prevention after the assessment of potential side-effects. As for the different associations of snus dipping and cigarette smoking with PAD risk, the difference in components of snus and tobacco may convey the clues that certain chemical components contained in tobacco or generated in cigarette smoking, such as arsenic^27^ and carbon monoxide^28^ but not nicotine elevate the risk of developing PAD.

There are several underlying mechanisms explaining the positive association between tobacco smoking and PAD risk. Smoking is associated with high risk of developing metabolic disorders, such as type 2 diabetes^29^, hypertension^5^, and obesity^5^, which are risk factors for PAD. In addition, smoking may increase PAD risk through systemic inflammation^30,31^, endothelial dysfunction^32^, and coagulation factors^33^. The null finding on the association between Swedish snuff dipping and PAD risk may be explained by limited peripheral arterial atherosclerosis effects of nicotine which is rich in snus products.

There are strengths and limitations of the present study. Strength includes a large sample size, a long follow-up period, and accurate diagnostic information from register. We used tobacco smoking as the positive control and a strong association between tobacco smoking and PAD risk validated the case definition. Snus dipping and tobacco smoking information was obtained from a self-reported questionnaire, which may lead to misclassification of the exposure. Over the follow-up period, individuals may change their tobacco and snus use, which may also introduce misclassification of the exposure. Even though we adjusted for important lifestyle factors in the analysis, we could not completely rule out the possibility that our results might be affected by residual confounding caused by unadjusted factors, like sleep features^34^ and mental factors^35^, although these factors have not been robustly associated with the development of PAD. This study included middle-aged and elderly men only. Thus, whether findings can be generalized to younger individuals and to women needs assessment. The power of the study was generally confined by a small number of PAD cases. In addition, we could not assess the association between Swedish snus dipping and PAD risk in never smokers also due to few cases (2 cases and 4 cases in past and current snus dippers in never smoker, respectively). However, any residual confounding from cigarette smoking cannot explain our null finding for snus dipping and PAD risk.

In conclusion, cigarette smoking but not Swedish snuff dipping likely increases the risk of PAD. Along with previous null findings on the associations of snus dipping with stroke and coronary heart diseases^13–16,19^, our findings indicate that Swedish snus dipping is unlikely to have an important impact on the development of vascular diseases.

## Data Availability

De-identified SIMPLER data are available for researchers upon application (http://www.simpler4health.se/). Data can be accessed by a reasonable request to the corresponding author.
